# Prevalence and correlates of caregiver burden among carers of older persons living with dementia in rural eastern Uganda

**DOI:** 10.1002/alz70858_096575

**Published:** 2025-12-24

**Authors:** Stephen Ojiambo Wandera, Monica M Diaz, Leah H Rubin, Noeline Nakasujja

**Affiliations:** ^1^ Makerere University, Kampala, Central, Uganda; ^2^ University of North Carolina at Chapel Hill School of Medicine, Chapel Hill, NC, USA; ^3^ Johns Hopkins University, Baltimore, MD, USA

## Abstract

There is limited data about caregiver burden among older persons in rural Uganda. This study aimed to determine the prevalence and correlates of caregiver burden among carers of older persons with dementia in rural eastern Uganda.

A cross‐sectional survey of **602 dyads of older persons and carers** was recruited. The **Zarit Burden Interview scale** was used to determine caregiver burden. Frequency distributions and logistic regressions were used to determine the prevalence and correlates of caregiver burden. The frequency of **caregiver burden was about 62%. About 44% reported moderate burden and 18% reported severe caregiver burden**. The correlates of caregiver burden will be determined shortly.

